# A Robust Transmission Scheduling Approach for Internet of Things Sensing Service with Energy Harvesting

**DOI:** 10.3390/s19143090

**Published:** 2019-07-12

**Authors:** Jie Hao, Jing Chen, Ran Wang, Yi Zhuang, Baoxian Zhang

**Affiliations:** 1 College of Computer Science and Technology, Nanjing University of Aeronautics and Astronautics, Nanjing 211106, China; 2 Collaborative Innovation Center of Novel Software Technology and Industrialization, Nanjing 210023, China; 3 School of Artificial Intelligence, University of Chinese Academy of Sciences, Beijing 100049, China

**Keywords:** energy harvesting, transmission scheduling, Internet of Things, robustness, uncertainty

## Abstract

Maximizing the utility under energy constraint is critical in an Internet of Things (IoT) sensing service, in which each sensor harvests energy from the ambient environment and uses it for sensing and transmitting the measurements to an application server. Such a sensor is required to maximize its utility under the harvested energy constraint, i.e., perform sensing and transmission at the highest rate allowed by the harvested energy constraint. Most existing works assumed a sophisticated model for harvested energy, but neglected the fact that the harvested energy is random in reality. Considering the randomness of the harvested energy, we focus on the transmission scheduling issue and present a robust transmission scheduling optimization approach that is able to provide robustness against randomness. We firstly formulate the transmission scheduling optimization problem subject to energy constraints with random harvested energy. We then introduce a flexible model to profile the harvested energy so that the constraints with random harvested energy are transformed into linear constraints. Finally, the transmission scheduling optimization problem can be solved traditionally. The experimental results demonstrate that the proposed approach is capable of providing a good trade-off between service flexibility and robustness.

## 1. Introduction

The Internet of Things (IoT) has drawn much attention due to its massive applications. One important IoT application is environment sensing, such as climate change sensing and water quality monitoring. In such IoT applications, a number of IoT devices equipped with sensors (we refer to the IoT devices as sensors hereafter) with wireless communication interfaces are deployed to sense the environment and report the readings to the application server. The communication network is typically a single-hop network with a star typology. Low-Power Wide Area Network (LPWAN) technologies including IEEE 802.11ah, Long Range (LoRa), SigFox, Narrow Band (NB)-IoT, and so on, can be used for single-hop communication. Recently, there has been growing concern about energy efficiency for IoT services [1]. Energy harvesting is one promising method to address this concern.

An energy-harvesting sensor can harvest ambient energy (e.g., solar, wind, thermal, vibration, etc.) to recharge its battery and thus is capable of maintaining an eternal lifetime. This allows sensors to be suitable to be deployed in tough circumstances. The main design objective for an energy-harvesting sensor is to fulfill its functionalities by adequately utilizing the harvested energy, i.e., achieve the maximum utility subject to the harvested energy constraint. The objective involves two main elements, utility and energy constraint. Utility can stand for a number of performance metrics, such as duty cycle [2,3], sensing coverage [4], throughput [5,6,7], data quality [8,9,10], decision fusion [11,12,13], and so on. The energy constraint regarding energy harvesting is well known as Energy Neutral Operation (ENO), that is the consumed energy of a sensor should not exceed the harvested energy after a finite horizon. In environment-sensing IoT applications, the main functionalities of a sensor are to sense environment and transmit the readings. The more readings transmitted, the higher sensing quality can be obtained. Therefore, we focus on the transmission scheduling design that aims to achieve the maximum data quality subject to the harvested energy constraint.

**Figure 1:**
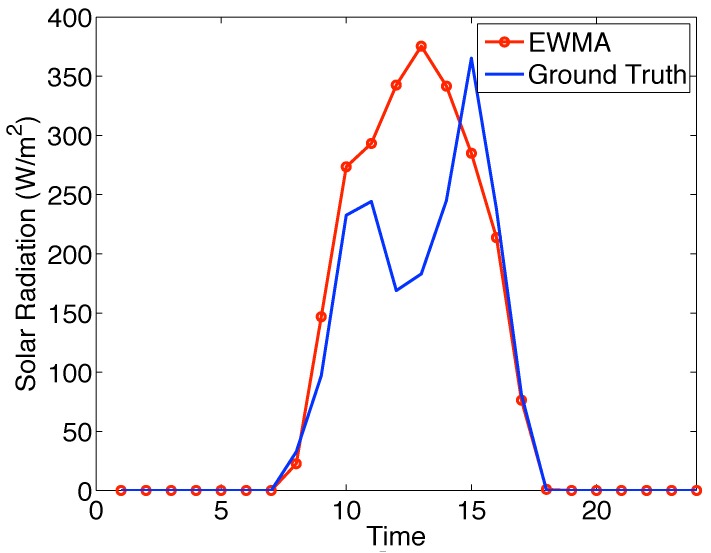
The energy model deviates from the ground truth. EWMA, Exponentially-Weighted Moving-Average.

However, there exists a big challenge in the transmission scheduling design, i.e., the unknown harvested energy profile. Although there has been extensive research on profiling the harvested energy, either predicting the harvested energy or modeling it as a random process, the harvested energy profile cannot be precise, and the impact of the inaccurate harvested energy profile on the performance has not been considered. Figure 1 shows the deviation of a solar energy model, Exponentially-Weighted Moving-Average (EWMA), proposed in [14] from the ground truth. The uncertainty in harvested energy makes the transmission scheduling design based on an exact energy model stray from the design objective. To improve the robustness against the harvested energy randomness, instead of performing transmission scheduling based on an exact harvested energy model, this paper presents an Uncertain Harvested Energy-based Transmission Scheduling approach (UHETS), which considers the harvested energy to be of a distribution fluctuating around an energy model. In this way, UHETS is able to achieve high robustness against the inaccuracy of the harvested energy model. The main contributions of this paper include:

- We formulate the transmission scheduling problem as a utility optimization problem subject to the energy constraints.
- We introduce a flexible model to describe the harvested energy, which is robust against the inaccuracy of an exact energy model.
- We conduct extensive experiments on real-world data and explore the impacts of the prediction model and various parameters; numerical results demonstrate the robustness of the proposed scheduling approach.

The remainder of this paper is organized as follows. Section 2 introduces related work. In Section 3 and Section 4, we present the proposed scheduling approach and adaptive transmission approach, respectively. Section 5 summarizes the presented approaches. The evaluation results are shown in Section 6 followed by the conclusion in Section 7.

## 2. Related Work

In this section, we introduce the related literature and focus on the interplay between the utility optimization and the energy profile. We categorize the related work into three classes.

### 2.1. Utility Optimization without Prior Knowledge of the Harvested Energy Profile

In this category, utility optimization is performed mainly according to the available energy. Vigorito et al. [14] aimed to optimize the average duty cycle and minimize the variance while maintaining ENO. They proposed a harvested energy model-free approach that kept track of the battery level instead of the harvested energy level and made adaptive duty cycle decisions based on adaptive control theory. Sharma et al. [7] claimed that the greedy policy that transmits the most data bits allowed by available energy had promising performance in terms of both throughput and mean delay. Yoon et al. [15] compared the harvested energy and its desired energy level and used the excess energy to compress and transmit data packets. As these approaches only considered the current available energy, they cannot guarantee global optimization. Djenouri et al. [16] treated the energy-harvesting sensor as an unlimited energy sensor and only used harvesting nodes as relay nodes to maintain network connectivity. The connectivity problem is equivalent to finding the minimum weighted connected dominating set in a vertex weighted graph. This assumption is impractical in many cases. For example, solar energy on a rainy day might be insufficient for full-time operation.

### 2.2. Utility Optimization Based on a Known Harvested Energy Profile

Simultaneous Wireless Information and Power Transfer (SWIPT), as a major type in this category, mainly addresses the resource allocation problem as an optimization problem subject to a energy harvesting model. The main effort is made to solve the optimization problem, for example to overcome the non-convexity of the optimization. Conventionally, the harvested energy is assumed as a linear function of input energy [17,18,19,20]. Based on the linear model, the energy/information efficiency is optimized, including the Rate-Energy (R-E) tradeoff in [17], the maximum achievable energy efficiency in [19], the users’ weighted sum uplink rate in [20], and the joint optimization of transmit power energy harvesting efficiency and interference power leakage-to-transmit power ratio in [18]. Vamvakas et al. [21] consider users’ actual needs in energy consumption during the wireless energy transfer phase and solved the optimization problem of each user’s utility function with respect to the optimal customized transmission power. However, the linear model is only applicable for the specific scenario in which the received powers are constant. In practice, the work in [22] showed that the wireless energy conversion efficiency varies with different input power energy levels. Based on the non-linear energy harvesting model, the resource allocation problem will be more complex [23,24,25,26]. In [23], the cross-layer multi-level optimization to maximize the power efficiency, user fairness, and channel non-reciprocity was studied. Boshkovska et al. [24] took the uncertainty of the Channel State Information (CSI) into account and addressed robust sum throughput maximization and maximization of the minimum individual throughput against imperfect CSI knowledge. The work in [25] aimed to minimize the transmit power in the scenario when multiple heterogeneous users existed. Xiong et al. [26] followed the work of [17], but derived the boundaries of the R-E region for MIMO broadcasting channels. For more literature surveys on SWIPT, please refer to [27].

Gunduz et al. [28] focused on offline transmission scheduling for both half-duplex and full-duplex two-hop communication scenarios and assumed the energy was harvested at known instants. Fan et al. [29] firstly used a known prediction model for the energy profile and modeled a multiple-hop network as a node-weighted space-time graph. Upon such a graph, the awake sensor node set with minimum cost was found to maintain the network connectivity. However, the simulation was conducted based on a randomly-generated topology instead of using an energy prediction model.

Maemoto et al. [30] considered the non-uniform harvesting rates for the sensor nodes and proposed a clustering control scheme, which consisted of adaptive cluster size control and adaptive backoff window control. EWMA [2] and its variances are widely used as (solar) energy prediction models. In EWMA, the predicted harvested energy is a weighted combination of historical data of previous days. Using EWMA, the work in [2] formulated the energy allocation problem as a duty cycle maximization problem subject to energy management constraints. Apparently, the assumption of the known harvested energy profile is not realistic since the energy harvesting process is naturally random.

### 2.3. Utility Optimization Assuming the Energy Harvesting Process as a Random Process

To address the uncertainty of harvested energy, some recent work modeled the harvested energy as a random process. The Markov chain is a promising tool for modeling a random process [31,32]. Tunc et al. [31] assumed a finite-state Continuous-Time Markov Chain (CTMC) model for characterizing the energy harvesting process and proposed an adaptive sensing policy that maximized the sensing rate of an IoT device while meeting the first battery outage probability constraint. Gorlatova et al. [33] treated the independent and identically distributed random harvested energy as a Markov decision process and developed an algorithm for calculating the maximum energy spending rate for a sensor node. The Markov model was applied for decentralized hypothesis testing [34] as well. In [34], the sensors made observations of a phenomenon and performed transmission scheduling to minimize the error probability of the hypothesis on the target phenomenon.

Ho et al. [6] aimed to maximize the throughput for point-to-point, flat-fading, single-antenna communication via power allocation. The energy harvesting and the time-varying wireless channel were modeled as a random process with a joint distribution to address their unpredictable nature. Huang et al. [35] assumed an energy harvesting model under which the energy arrival time and the harvested amount were known prior to transmissions and addressed the throughput maximization problem under the Gaussian relay channel. Yadav et al. [36] focused on the problem that the randomness and arrivals of harvested energy may induce interruptions to single-hop communications. To overcome the interruptions, the work in [36] assumed that the arrival of harvested energy followed a Bernoulli random process and proposed an adaptive retransmission scheme. The retransmission scheme can highly improve the communication reliability against the uncertainty of harvested energy. Aman et al. [2] modeled the solar energy profile by using the average harvesting rate and lower/upper bound. In order to guarantee ENO, the lower bound of harvested energy was used to maximize the duty cycle of a sensor node. This lower bound-based guarantee would incur inefficient energy usage and degraded utility. Chamanian et al. [37] focused on vibration energy and maximized the duty cycle subject to ENO. Hao et al. [3] adopted a robust energy model in which the harvested energy fluctuated around a reference distribution to pursue the maximum duty cycle. However, they did not consider the fact that the uncertainty of cumulative harvested energy might change, and their proposed duty cycle maximization approach may result in an overly strict energy maintaining policy. Although the randomness of harvested energy was considered in the above work, the impact of the deviation of a hypothetical random energy model from the ground truth on the network performance was not studied.

## 3. Robust Transmission Scheduling

This section will describe how to formulate the transmission scheduling problem and how to solve it in detail.

### 3.1. Network Model

Consider that in an environment sensing IoT application, each sensor equipped with an energy-harvesting battery with a non-ideal energy buffer senses a random field and generates packets to be transmitted to the application server.

Time is slotted, and each slot lasts for ΔT, while one decision cycle lasts for *T*. During the *t*th slot, *n* samples are supposed to be taken and transmitted by the sensor. However, due to the energy constraint, only a subset of the samples, i.e., n(t)<n samples, is eligible for sensing and transmission in the *t*th slot. This can be considered as a matrix completion problem. Given an original n×T data sample matrix $M_{n\times T}$, for each slot *t*, only n(t) samples are transmitted to the application server. That is, the transmission scheduling decides an indicator matrix $\Omega_{n\times T}$, and each entry Ω(i,j) denotes whether or not the corresponding entry in $M_{n\times T}$ is selected for transmission. If the entry in location (i,j) is selected: Ω(i,j)=1, otherwise, Ω(i,j)=0. Thus, the received data matrix by the application server is:

O=Ω•M,

where • denotes the Hadamard product (the element-wise product of two matrices). Upon the received matrix $O_{m\times n}$, the application server then utilizes a matrix completion algorithm to reconstruct a complete matrix $\tilde{M}$. Obviously, the transmission scheduling scheme has a big impact on data recovery quality. Generally speaking, the more transmitted samples, the higher the recovery quality. Therefore, we aimed to design a transmission scheduling scheme that leads to a high recovery quality. The design of the matrix completion method is out of the scope of our work. In this paper, we just adopt a classic algorithm, Singular-Value Thresholding (SVT) [38], as the matrix completion method. Considering that the energy cost for computation is negligible compared with that for communication and sensing (for energy-hungry sensors) [39], we assume that sensing and transmission consume most of the energy in a sensor node and ignore the other consumption (The proposed approach is suitable for the sensor nodes whose energy cost for sensing is trivial. In such cases, the sensor node can sense the environment at a high rate, but only transmits a subset of the sensing readings based on the proposed transmission scheduling.).

Each sensor node in the network has finite battery capacity $E_{max}$. Let $E_{t}$ denote the remaining energy of the battery at slot *t*, $E_{h}\left(t\right)$ denote the harvested energy during slot *t*, and $E_{c}$ denote the energy cost for single-packet sensing and transmission. Given $E_{c}$, $E_{h}\left(t\right)$ at slot *t*, then at slot *t*, the energy buffer will be updated as:

(1) $$\begin{array}{cc} & E_{t}=E_{0}-\sum_{i=1}^{t}n\left(i\right)E_{c}+\sum_{i=1}^{t}E_{h}\left(i\right), \end{array}$$

with the constraints:

(2) $$\begin{array}{cc} 0\leq E_{t} & \forall t\in [0,T] \end{array}$$

(3) $$\begin{array}{cc} E_{t}\leq E_{max} & \forall t\in [0,T] \end{array}$$

(4) $$\begin{array}{c} n(t)\leq n. \end{array}$$

Moreover, ENO imposes an additional constraint that over a decision period *T*, the sensor node is expected to be energy neutral. In other words, the remaining energy of a sensor node should be higher than the initial energy. This can be formulated as:

(5) $$\begin{array}{c} E_{T}\geq E_{0}, \end{array}$$

where *T* is generally set to be a twenty-four-hour duration if we adopt the solar energy source.

The variables used in this paper are summarized in Table 1.

### 3.2. Optimization Formulation

In this paper, we aim to optimize the transmission scheduling and further optimize the data recovery quality subject to the energy constraints over the time horizon. To this end, we hoped that the overall transmission number could be as high as possible. The more samples transmitted, the higher the data quality a sensor node can provide. Moreover, we also desired fairness among all slots, that is we absolutely did not expect n(t)=0 for slot *t*. In the case of a solar harvesting sensor node, there is no harvested energy during the night, which might lead to zero transmissions during the night. Obviously, this will lead to extreme high information loss during the night. Motivated by this, the optimization problem of transmission scheduling subject to the energy constraints is defined as follows:

(6) $$\begin{array}{cc} \max & sum(N)-var(N)\\ s.t.\; & \left(1),(2),(3),\left(4\right),(5\right) \end{array}$$

where N=[n(1),n(2),⋯,n(T)], sum denotes the summation operation, and var is the variance of N. We believe this optimization objective will yield high data reconstruction quality by using matrix completion.

### 3.3. Handling the Random Variable

As illustrated in Section 1, the constraints involve a random variable $E_{h}\left(t\right)$, which brings in a big challenge to solve the optimization problem (6). In this subsection, we shall illustrate how to handle the uncertainty of $E_{h}\left(t\right)$ in detail.

For a constraint with a random variable, a robust way to guarantee the constraint is to guarantee the worst case probability. For example, if we have a constraint x>y where *y* is a random variable uniformly distributed within [0,1], the worst case for x>y is when y=1. Therefore, if we replace *y* with its upper bound of one, we can guarantee x>y with a probability of 100%. However, in most applications, it is too strict or infeasible to guarantee 100%. We can approximately guarantee the original constraint with a probability of 1−ϵ, where ϵ∈[0,1]. In this case, we can firstly calculate a threshold $y^{u}$ such that $P(y^{u}\geq y)=1-\epsilon$ and then replace x>y with $x>y^{u}$. The original constraint is transformed into a traditional linear constraint.

Likewise, we can tackle $E_{h}\left(t\right)$ in the same way. However, we notice that our problem is different from the example in two aspects. One is that $E_{t}$ involves the sum of $E_{h}\left(i\right),1\leq i\leq t$. It is too strict to guarantee the worst case probability for every $E_{h}\left(i\right),t\in \left[1,t\right]$ considering that the sum of $E_{h}\left(i\right)$ will compensate the other, especially when *t* is large enough. The other is that the distribution of the random variable in our problem is not known. We had to use a novel model to obtain the threshold and transform the original constraints to traditional linear constraints. To address these two difficulties, we introduced a new variable $S_{h}\left(t\right)=\sum_{i=1}^{t}E_{h}\left(i\right)$ and explored the threshold regarding $S_{h}\left(t\right)$.

#### 3.3.1. Transforming the Constraints

We firstly processed the constraint $E_{t}\geq 0$ in Constraint (2). Constraints $E_{t}\leq E_{max}$ in (3) and $E_{T}\geq E_{0}$ in (5) can be transformed in the same way. The transformation processes of Constraints (3) and (5) are omitted here due to space limitations.

With $S_{h}\left(t\right)$, Equation (1) can be transformed to:

(7) $$\begin{array}{c} E_{t}=E_{0}-\sum_{i=1}^{t}n\left(i\right)E_{c}+S_{h}\left(t\right). \end{array}$$

From $E_{t}\geq 0$, we have:

(8) $$\begin{array}{c} S_{h}\left(t\right)\geq \sum_{i=1}^{t}n\left(i\right)E_{c}-E_{0}. \end{array}$$

We let $s_{h}\left(t\right)=\sum_{i=1}^{t}n\left(i\right)E_{c}-E_{0}$ and then obtain:

(9) $$\begin{array}{c} S_{h}\left(t\right)\geq s_{h}\left(t\right). \end{array}$$

This constraint can be guaranteed with a probability of (higher than) 1−ϵ by:

(10) $$\begin{array}{c} S_{h}^{u}\left(t\right)\geq s_{h}\left(t\right) \end{array}$$

(11) $$\begin{array}{c} P(S_{h}\left(t\right)\geq S_{h}^{u}\left(t\right))=1-\epsilon , \end{array}$$

where P(*) denotes the probability of condition * and ϵ∈[0,1] is a sufficiently small value. Since the distribution of $S_{h}\left(t\right)$ is unknown, it is difficult to derive the exact value of $P(S_{h}\left(t\right)\geq S_{h}^{u}\left(t\right))$. Therefore, in order to satisfy Constraint (9) with a probability of (higher than) 1−ϵ, we should guarantee Condition (9) regardless of $f(S_{h}\left(t\right))$ by guaranteeing:

(12) $$\begin{array}{cc} & \min_{f(S_{h}\left(t\right))}P\left(S_{h}\left(t\right)\geq S_{h}^{u}\left(t\right)\right)=1-\epsilon , \end{array}$$

where $f(S_{h}\left(t\right))$ is the ground truth Probability Density Function (PDF) of $S_{h}\left(t\right)$. In this paper, we use the PDF and distribution interchangeably. We can see that ϵ indicates the robustness level against the uncertainty, that is we can adapt the robustness by tuning ϵ.

In the next step, we need to cope with the unknown $f(S_{h}\left(t\right))$ and calculate the threshold $S_{h}^{u}\left(t\right)$ to satisfy Equation (12).

#### 3.3.2. Calculating Threshold $S_{h}^{u}\left(t\right)$

**Figure 2:**
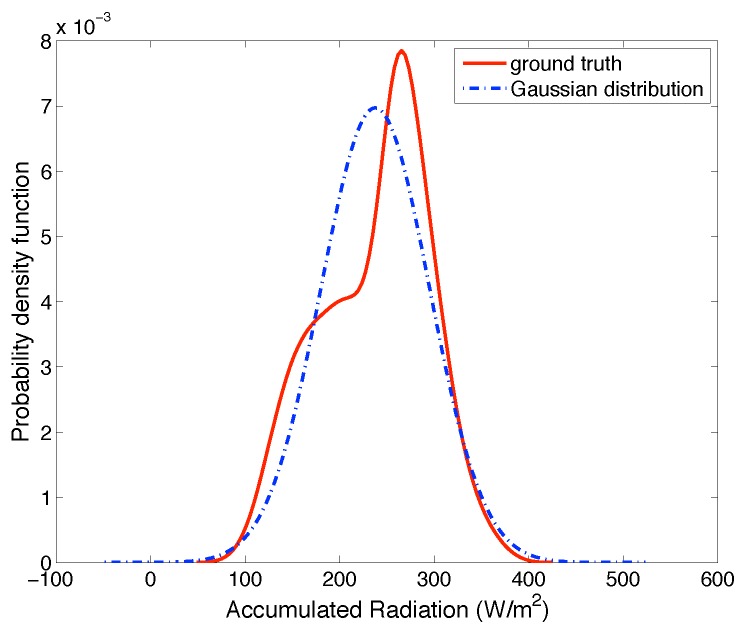
The probability density of $S_{h}\left(10\right)$ is close to a Gaussian distribution.

**Figure 3:**
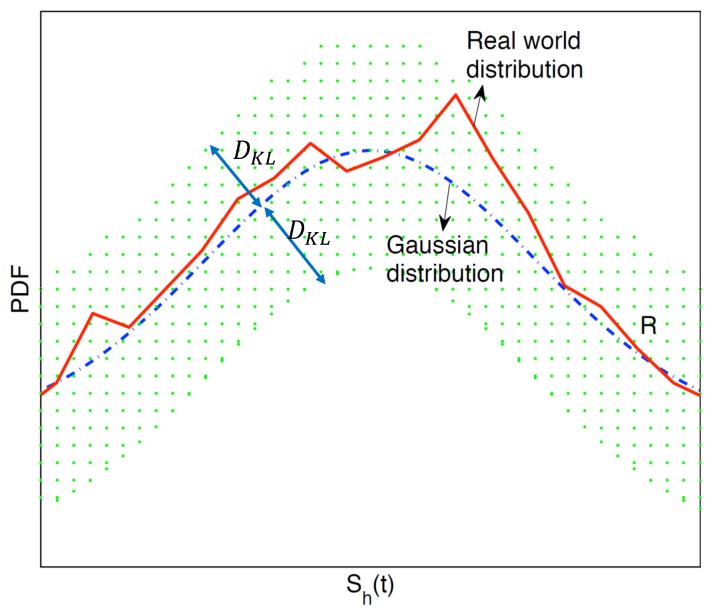
The formulation of $f(S_{h}\left(t\right))$. The red solid curve is the ground truth PDF, which is unknown in practice. The blue dotted curve is an empirical Gaussian distribution. The dotted area denotes R in which each distribution has a distance from the Gaussian distribution lower than a limit $D_{KL}$. Since the ground truth distribution is close to the Gaussian distribution, we say that the ground truth distribution belongs to R. Please note that this figure is for illustrative purposes only.

Although there is no precise prediction model or distribution model to describe $S_{h}\left(t\right)$, $S_{h}\left(t\right)$ can be considered to be close to a prediction, and its probability density is close to a known distribution [40]. The former can be demonstrated in Figure 1, in which EWMA can provides a close estimate. Figure 2 shows that the PDF of $S_{h}\left(t\right)$ was close to a Gaussian distribution [40,41]. The data used in Figure 2 were the values of $S_{h}\left(10\right)$ (i.e., the accumulative harvested energy until 10:00) in January from 2001–2019. We omitted the other results since they performed similarly. Therefore, we assumed that the real-world distribution of $S_{h}\left(t\right)$ was close to a Gaussian distribution in which the mean value was a prediction based on some model (e.g., EWMA) and the variance was obtained empirically based on historical data. Moreover, the Kullback–Leibler divergence (KL divergence) between the Gaussian distribution and real-world distribution of $S_{h}\left(t\right)$ for every slot was only about 0.1. Thus, we can use the Gaussian distribution along with its difference from the ground truth to describe $S_{h}\left(t\right)$. In this way, the ground truth distribution of $S_{h}\left(t\right)$ belongs to a domain R,

(13) $$\begin{array}{cc} & R=\{f\left(S_{h}\left(t\right)\right)|Distance\;between\;\\ & f\left(S_{h}\left(t\right)\right)\;and\;g\left(S_{h}\left(t\right)\right)\;is\;less\;than\;a\;limit\}, \end{array}$$

where $g(S_{h}\left(t\right))$ is the empirical Gaussian distribution based on a prediction model. Figure 3 is an illustrative figure to show how we formulated the unknown $f(S_{h}\left(t\right))$. In Figure 3, as we assumed the ground truth distribution was close to a Gaussian distribution, it lied within the dotted region R in which each distribution had a distance from the Gaussian distribution lower than a limit $D_{KL}$.

The distance between the two distributions can be calculated by the KL divergence, which can be formulated as:

(14) $$\begin{array}{c} E_{f}\left[\ln f\left(S_{h}\left(t\right)\right)-\ln g\left(S_{h}\left(t\right)\right)\right]=\int_{0}^{+\infty }\left[\ln f\left(S_{h}\left(t\right)\right)-lng\left(S_{h}\left(t\right)\right)\right]f\left(S_{h}\left(t\right)\right)dS_{h}\left(t\right), \end{array}$$

where $E_{f}\left(*\right)$ denotes the integral of expression * with $f(S_{h}\left(t\right))$. As a result, R can be formulated as:

(15) $$\begin{array}{cc} & R=\{f\left(S_{h}\left(t\right)\right)|E_{f}\left[\ln f\left(S_{h}\left(t\right)\right)-\ln g\left(S_{h}\left(t\right)\right)\right]\leq D_{KL}\}, \end{array}$$

where $D_{KL}$ is the distribution difference limit. The more uncertainty in $S_{h}\left(t\right)$, the less confidence on the Gaussian distribution, and thus, the larger $D_{KL}$.

Therefore, with $f(S_{h}\left(t\right))\in R$, to obtain $S_{h}^{u}\left(t\right)$ satisfying Equation (12) to obtain $S_{h}^{u}\left(t\right)$ such that:

(16) $$\begin{array}{c} \min_{f(S_{h}\left(t\right))\in R}P\left(S_{h}\left(t\right)\geq S_{h}^{u}\left(t\right)\right)=1-\epsilon , \end{array}$$

which is further equivalent to:

(17) $$\begin{array}{c} \max_{f(S_{h}\left(t\right))\in R}P\left(S_{h}\left(t\right)<S_{h}^{u}\left(t\right)\right)=\epsilon . \end{array}$$

We denoted the left part of Equation (17) as $P_{m}\left(S_{h}^{u}\left(t\right)\right)$ and divided the solution of Constraint (17) into two subproblems, which were calculating $P_{m}\left(S_{h}^{u}\left(t\right)\right)$ as the function of variable $S_{h}^{u}\left(t\right)$ and calculating $S_{h}^{u}\left(t\right)$ such that $P_{m}\left(S_{h}^{u}\left(t\right)\right)=\epsilon$. We first solved the first subproblem, which can be formalized as:

(18) $$\begin{array}{c} P_{m}\left(S_{h}^{u}\left(t\right)\right)=\max_{f(S_{h}\left(t\right))\in R}P\left(S_{h}\left(t\right)<S_{h}^{u}\left(t\right)\right)=\max_{f(S_{h}\left(t\right))\in R}\int_{0}^{S_{h}^{u}\left(t\right)}f\left(S_{h}\left(t\right)\right)dS_{h}\left(t\right), \end{array}$$

(19) $$\begin{array}{cc} s.t. & E_{f}\left[\ln f\left(P_{h}\left(t\right)\right)-\ln g\left(P_{h}\left(t\right)\right)\right]\leq D_{KL} \end{array}$$

(20) $$\begin{array}{c} E_{f}\left[1\right]=1. \end{array}$$

In order to avoid the variable in the limits of the integral, $P_{m}\left(S_{h}^{u}\left(t\right)\right)$ in Equation (18) can also be transformed to:

(21) $$\begin{array}{cc} \max_{f(S_{h}\left(t\right))\in R} & \int_{0}^{+\infty }w\left(S_{h}\left(t\right),S_{h}^{u}\left(t\right)\right)f\left(S_{h}\left(t\right)\right)dS_{h}\left(t\right), \end{array}$$

where $w(S_{h}\left(t\right),S_{h}^{u}\left(t\right))$ is an auxiliary function as:

(22) $$\begin{array}{c} w\left(S_{h}\left(t\right),S_{h}^{u}\left(t\right)\right)=\left\{\begin{array}{cc} 0 & S_{h}\left(t\right)\geq S_{h}^{u}\left(t\right);\\ 1 & S_{h}\left(t\right)<S_{h}^{u}\left(t\right). \end{array}\right. \end{array}$$

Fortunately, the problems (19)–(21) is convex. The proof of the convexity is shown in Appendix A. Based on the convexity of the optimization problem and Slater’s condition, we can utilize the duality to solve (18)–(20). By applying the Lagrangian method, we can obtain $P_{m}\left(S_{h}^{u}\left(t\right)\right)$ as follows:

$$\begin{array}{cc} P_{m}\left(S_{h}^{u}\left(t\right)\right)= & \min_{\alpha ,\beta }\max_{f(S_{h}\left(t\right))}E_{f}[w\left(S_{h}\left(t\right),S_{h}^{u}\left(t\right)\right) \end{array}$$

(23) $$\begin{array}{c} -\alpha \left(E_{f}\left[\ln f\left(P_{h}\left(t\right)\right)-\ln g\left(P_{h}\left(t\right)\right)\right]-D_{KL}\right)-\beta \left(E_{f}\left[1\right]-1\right) \end{array}$$

(24) $$\begin{array}{cc} = & \min_{\alpha ,\beta }\max_{f(S_{h}\left(t\right))}E_{f}\left[w\left(S_{h}\left(t\right),S_{h}^{u}\left(t\right)\right)-\beta -\alpha \ln\frac{f(S_{h}\left(t\right))}{g(S_{h}\left(t\right))}\right]+\alpha D_{KL}+\beta , \end{array}$$

where α≥0 and β are Lagrangian multipliers associated with Constraints (19) and (20), respectively. The optimal distribution function $f^{*}\left(S_{h}\left(t\right)\right)$ and $\alpha^{*},\beta^{*}$ for the optimization problem (23) can be derived as in Appendix B in detail. We only present the derivation results here. The optimal distribution function is:

(25) $$\begin{array}{c} f^{*}\left(S_{h}\left(t\right)\right)=g\left(S_{h}\left(t\right)\right)\exp\left(\frac{w\left(S_{h}\left(t\right),S_{h}^{u}\left(t\right)\right)-\beta^{*}}{\alpha^{*}}-1\right), \end{array}$$

where $\alpha^{*},\beta^{*}$ are obtained by solving the following.

(26) $$\begin{array}{c} U\left(S_{h}^{u}\left(t\right)\right)e^{\beta^{*}/\alpha^{*}}+V\left(S_{h}^{u}\left(t\right)\right)e^{\left(1-\beta^{*}\right)/\alpha^{*}}=0, \end{array}$$

(27) $$\begin{array}{c} V\left(S_{h}^{u}\left(t\right)\right)e^{\left(1-\beta^{*}\right)/\alpha^{*}}-\beta^{*}-\alpha^{*}\left(1+D_{KL}\right)=0, \end{array}$$

where $U\left(S_{h}^{u}\left(t\right)\right)=G\left(S_{h}^{u}\left(t\right)\right)e^{-1}$, $V\left(S_{h}^{u}\left(t\right)\right)=\left(1-G\left(S_{h}^{u}\left(t\right)\right)\right)e^{-1}$, and G(*) is the cumulative distribution of $g(S_{h}\left(t\right))$.

With the obtained $\alpha^{*},\beta^{*}$, we then solved the second subproblem, i.e., calculating the threshold $S_{h}^{u}\left(t\right)$ from $P_{m}\left(S_{h}^{u}\left(t\right)\right)=\epsilon$.

However, it is difficult to calculate an explicit solution of $S_{h}^{u}\left(t\right)$ and $\alpha^{*},\beta^{*}$ by solving Equations (26) and (27). A heuristic algorithm to calculate $S_{h}^{u}\left(t\right)$ and $\alpha^{*},\beta^{*}$ is required. We note that $P_{m}\left(S_{h}^{u}\left(t\right)\right)$ is non-decreasing regarding $S_{h}^{u}\left(t\right)$, and a binary search method is presented in Algorithm A1 in Appendix C to search for proper solutions.

## 4. Adaptive Transmission Scheduling

UHETS can be performed based on a 24-h solar energy prediction just once per day. As the real-world harvested energy values may vary from the predicted values, we can adapt the transmission scheduling based on the observed harvested energy. One method is to update the predicted energy profile in real time and perform UHETS every slot, which is computationally consumptive, obviously. Another alternative is to adaptively tune the transmission scheduling in a more lightweight way. The basic idea is if $S_{h}\left(t\right)>S_{h}^{\prime }\left(t\right)$, where $S_{h}\left(t\right)$ is the observed harvested energy and $S_{h}^{\prime }\left(t\right)$ is the predicted value, the extra energy can be used to increase the transmissions of the latter slots. Otherwise, the transmissions of the latter slots should be decreased to some extent accordingly.

As we hoped to maximize the objective sum(N)−var(N), if the excessive energy or energy shortage was fixed, the change in sum(N) was fixed as well as $|\left(S_{h}\left(t\right)-S_{h}^{\prime }\left(t\right)\right)|/E_{c}$. Maximizing sum(N)−var(N) now becomes minimizing var(N), which desires N to be more even by adjusting the transmission scheduling. The detailed procedure of the adaptive transmission scheduling algorithm based on UHETS (UHETS-adaptation) is presented in Algorithm 1. We take an example to explain the working procedure.

**Algorithm 1** UHETS-adaptation**Input:**ct, N=[n(ct+1),⋯,n(T)], $S_{h}\left(ct\right)$, $S_{h}^{\prime }\left(ct\right)$ **Output:** Adjusted transmission decision for the remaining slots, i.e., N=[n(ct+1),⋯,n(T)].
1:$N_{a}$ is sorted N in the ascending order of the number of samples transmitted.2:$Q_{a}$ is the index of $N_{a}$.3:$N_{d}$ is sorted N in the descending order of the number of samples transmitted.4:$Q_{d}$ is the index of $N_{d}.$5:**if**$S_{h}\left(t\right)>S_{h}^{\prime }\left(t\right)$**then** //If the harvested energy is more than the expectation6:  $\Delta S_{h}=S_{h}\left(t\right)-S_{h}^{\prime }\left(t\right)$ // excessive energy7:  $\Delta n=\Delta S_{h}/E_{c}$ // Δn need to be added to N in total8:  **for** k=1→T−ct **do**9:   $Avg=(\sum_{j=1}^{k}\left(N_{a}\left(k\right)+\Delta n\right)/k$
10:   **if** $N_{a}\left(k+1\right)<Avg$ **then** // if involving $N_{a}\left(k+1\right)$ would make N more even11:    continue; // involve $N_{a}\left(k+1\right)$ in the adjustment12:   **else** // adjust $N_{a}\left(1\right)$ to $N_{a}\left(k\right)$13:    **for** j=1→k **do**14:     $N_{a}\left(j\right)=floor\left(Avg\right)$ //assign $N_{a}\left(1\right)$ to $N_{a}\left(k\right)$ uniformly15:    **end for**16:    $Residual=mod(\sum_{j=1}^{k}N_{a}\left(j\right)+\Delta n,k)$
17:    **for** j=k→k−Residual **do**18:     $N_{a}\left(j\right)=N_{a}\left(k\right)+1$ //assign the residual transmissions19:    **end for**20:    **for** k=1→T−ct **do**21:     $n\left(Q_{a}\left(k\right)\right)=N_{a}\left(k\right)$ //restore the order of N22:    **end for**23:    break;24:   **end if**25:  **end for**26:**else** // if the harvested energy is less than the expectation27:  $\Delta S_{h}=S_{h}^{\prime }\left(t\right)-S_{h}\left(t\right)$ // energy shortage28:  $\Delta n=\Delta S_{h}/E_{c}$ //Δn need to be deduced from *N* in total29:  **for** k=1→T−ct **do**30:   $Avg=(\sum_{j=1}^{k}n\left(Q_{d}\left(j\right)\right)-\Delta n)/k$
31:   **if** $N_{d}\left(k+1\right)>Avg$ **then** //if involving $N_{d}\left(k+1\right)$ would make N more even32:    continue; //involve $N_{d}\left(k+1\right)$ in the adjustment33:   **else** //adjust $N_{d}\left(1\right)$ to $N_{d}\left(k\right)$ evenly34:    $Residual=mod(\sum_{j=1}^{k}n\left(Q_{d}\left(j\right)\right)-\Delta n,k)$
35:    **for** j=1→k **do**36:     $N_{d}\left(j\right)=ceil\left(Avg\right)$
37:    **end for**38:    **for** j=k+1−Residual→k **do**39:     $N_{d}\left(j\right)=N_{d}\left(j\right)+1$
40:    **end for**41:    **for** k=1→T−ct **do**42:     $n\left(Q_{d}\left(k\right)\right)=N_{d}\left(k\right)$ //restore the order of N43:    **end for**44:    break;45:   **end if**46:  **end for**47:**end if**


At Slot 19 (ct=19, where ct means the current slot), we observed that more energy was harvested than expectation, and Δn=4 transmissions should be added in the subsequent slots (from Slots 20–24). The original transmission decision for the subsequent slots was N=[4,8,1,1,1]. In order to decrease var(N), a straightforward way is to utilize four to make the elements in N as even as possible.

We firstly sorted *N* in an ascending order as $N_{a}=\left[1,1,1,4,8\right]$ as shown in Line 1 in Algorithm 1. Starting from one (k=1 in Line 8), we decided if we should add four to $N_{a}\left(1\right)$ or evenly distribute four to $N_{a}\left(1\right)$ and $N_{a}\left(2\right)$ by comparing if $N_{a}\left(2\right)<\left(N_{a}\left(1\right)+4\right)/1$. Obviously, the latter will deduce a less var(N) if $N_{a}\left(2\right)<\left(N_{a}\left(1\right)+4\right)/1$. We should involve $N_{a}\left(2\right)$ in the adjustment. That is, Lines 10–11 were triggered, and we proceeded to Line 8 with k=2. Next, we decided if we should involve $N_{a}\left(3\right)$. As $N_{a}\left(3\right)<\left(N_{a}\left(1\right)+N_{a}\left(2\right)+4\right)/2$, Lines 10–11 were triggered the second time, and we proceeded to Line 8 with k=3. At this time, we found $N_{a}\left(4\right)>\left(N_{a}\left(1\right)+N_{a}\left(2\right)+N_{a}\left(3\right)+4\right)/3$, which means involving $N_{a}\left(4\right)$ did not help with reducing var(N). Therefore, now, we should execute Lines 13–23 to distribute Δn=4 to $N_{a}\left(1\right),N_{a}\left(2\right)$, and $N_{a}\left(3\right)$ as evenly as possible. As the summary of $N_{a}\left(1\right),N_{a}\left(2\right)$, and $N_{a}\left(3\right)$ is fixed as $\sum_{j=1}^{3}N_{a}\left(j\right)+\Delta n=7$. In order to distribute seven to the three elements uniformly, Lines 13–15 firstly assign floor(Avg)=floor(7/3)=2 to all three elements, and Residual=1 is left for assignment. Lines 16–19 assign Residual=1 to Residual=1 elements, one for each element, and $N_{a}\left(1\right)=2+1=3$. Finally, N=[4,8,3,2,2] is obtained. The procedure of adjustment in the case of energy shortage is similar, which is omitted.

## 5. Summary and Discussion

In this section, we will firstly summarize the working procedure of UHETS and then discuss the scalability and complexity.

### 5.1. Working Procedure Summary

The overall working procedure of UHETS is summarized in Algorithm 2. Firstly, $D_{KL}$ and ϵ are pre-set according to the application service, and σ as the variance of $S_{h}\left(t\right),t\in \left[1,T\right]$ is obtained based on historical data. At the beginning of every decision cycle, as shown in Lines 2–3, we firstly updated $g(S_{h}\left(t\right))$ for this decision cycle by using a prediction model (e.g., EWMA [2]). As we chose T=24 for solar energy, in this case, we firstly updated $g(S_{h}\left(t\right)),t\in \left[1,T\right]$ of the incoming day using an existing prediction model. Consequently, Lines 4–5 transform the original constraints in the optimization problem (6) to linear constraints. Using the linear constraints, Line 6 solves (6) and obtains the transmission scheduling N=[n(1),…,n(T)]. Upon the transmission scheduling, at each slot, the transmission scheduling is adjusted depending on the observed harvested energy by using UHETS-adaptation, as shown in Lines 7–9. Finally, the transmission scheduling is carried out, and matrix completion is used to recover the original samples. 

**Algorithm 2** Working procedure of UHETS**Input:**$D_{KL}$ and ϵ required by the application, the variance of $S_{h}\left(t\right),t\in \left[1,24\right]$ based on historical data denoted as σ **Output:**
N=[n(1),…,n(T)]1:**for** each decision cycle (every day) **do**2:  Calculate the harvested energy prediction of $S_{h}\left(t\right),t\in \left[1,24\right]$ as ${\hat{S}}_{h}\left(t\right)$ using a prediction model3:  Update the Gaussian distribution of $S_{h}\left(t\right),t\in \left[1,24\right]$ as $g\left(S_{h}\left(t\right)\right)=N\left({\hat{S}}_{h}\left(t\right),\sigma \right)$4:  Calculate $S_{h}^{u}\left(t\right),t\in \left[1,24\right]$ using Algorithm A15:  Replace $S_{h}\left(t\right)$ with $S_{h}^{u}\left(t\right)$ in the original constraints in (6) so that the constraints become linear6:  Solve the optimization problem (6) and obtain the transmission transmission scheduling N7:  **for** slot←1:T **do**8:   Perform UHETS-adaptation to adjust N for each slot9:  **end for**10:**end for**


### 5.2. Scalability

The proposed UHETS was designed for a single-hop star-topology network in which each sensor node independently decides its sensing and transmission scheduling. Although we describe UHETS only using one sensor node and one application server, UHETS is suitable for a multi-sensor network inherently. As UHETS does not involve multihop routing or cooperation among multiple sensor nodes, it inherently accommodates mobility [42]. For the same reason, UHETS is also suitable for clustered networks [43]. However, for a clustered network, cooperation among cluster members can be applied to further improve the data quality subject to the energy constraints.

In this paper, we do not consider the transmission failure caused by interference or channel failure. Retransmissions upon transmission failure apparently influence the transmission scheduling. However, matrix completion adopted in UHETS will alleviate the impact of transmission failure since it only requires the reception of sufficient data packets. As a result, retransmissions can be depressed if sufficient data packets are transmitted successfully. The detailed transmission implementation considering interference and retransmission scheme will be explored in future work.

### 5.3. Complexity Analysis

The complexity of UHETS contains three main components, the complexity of transforming the original constraints to the linear constraints, i.e., the complexity of Algorithm A1, the complexity to solve the convex Quadratic Programming (QP) optimization problem with linear constraints, and the complexity of UHEST-adaption in Algorithm 1.

**Figure 4:**
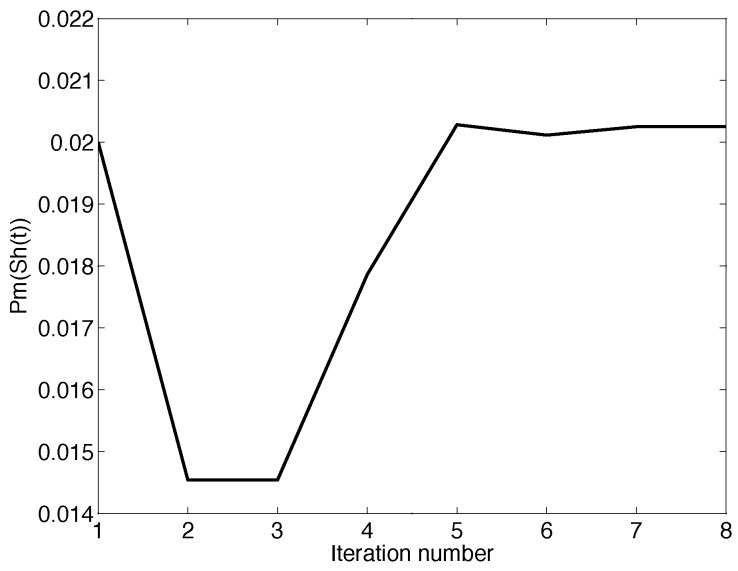
The convergence of Newton’s method.

Algorithm A1 has two loops, the outer loop being a binary search and the inner loop Newton’s method. The former has a complexity of $O(log_{2}\left(\gamma /\rho \right))$, while it would make more sense to discuss the convergence regarding Newton’s method. In Figure 4, we randomly choose one decision cycle in January 2018 and plot the convergence time of Newton’s method in this decision cycle. As seen in the figure, Newton’s method can converge in less than 10 iterations. In fact, Newton’s method can always converge in about 10 iterations in every decision cycle.

**Figure 5:**
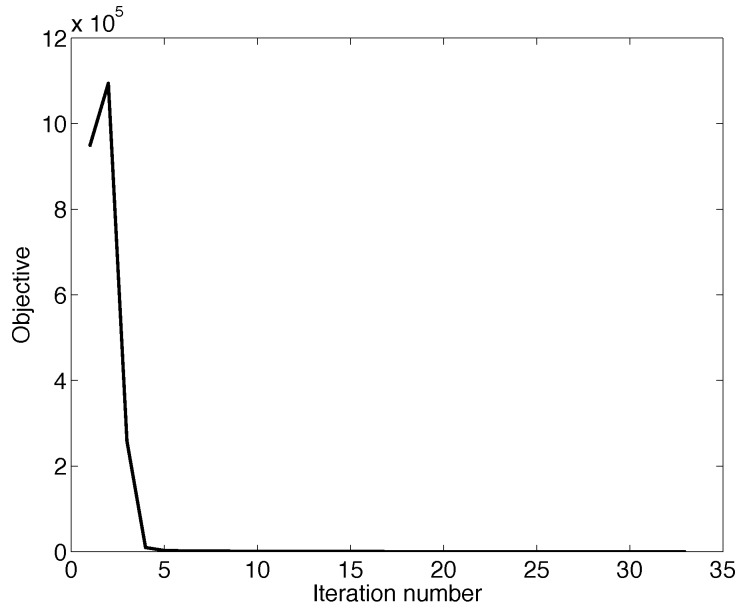
The convergence of the QP method.

When solving the convex QP problem, considering that integer optimization requires relatively high computational overhead and the transmission scheduling in this paper is for low-cost embedded systems, we removed the integer constraint of N when implementing the presented transmission scheduling and rounded down the resulting N to obtain the final scheduling scheme. The best-known running time if the interior point method is used for convex QP is $O(m^{1/2}L)$ iterations, where *L* is the length of input data in Turing machine theory and m=3T+1 (T=24) is the number of constraints. However, the practical convergence is more optimistic. The average iteration number was about 30. An example of the convergence in one randomly-chosen decision cycle is shown in Figure 5.

In Algorithm 1, the sorting process in Lines 1–4 is dominant regarding complexity. Since Algorithm 1 is performed every slot, the overall complexity is $O(T^{3})$, which is trivial for one decision cycle (one day).

In general, as UHETS has a limited scale, the computational complexity is acceptable for low-cost IoT sensors. In order to present the running time of UHETS, we also implemented UHETS using CVXGEN [44], an open-source optimization solver, on a low-cost MCU, STM32L4. For each decision cycle (T=24), UHETS took roughly 900 ms at a current of 8 mA and voltage of 3.6 V. Considering that the optimization is solved only once everyday, this cost is acceptable for this ultra-low-cost MCU.

## 6. Evaluation

In the evaluation, we supposed that in an environment sensing IoT application, each sensor senses the solar radiation and transmits the readings via the NB-IoT communication interface. We used the data of solar radiation spanning from 1 January 2001 to 31 December 2018 as the historical data for obtaining empirical parameters and the data from 1 January 2018–31 March 2018 for performance evaluation. The solar radiation data are published in units of W/m${}^{2}$ by NREL (National Renewable Energy Laboratory) [45]. The solar panel size was assumed as 10 cm × 10 cm, and the charging efficiency was 0.7 [4]. The harvested energy was the product of solar irradiation, solar panel size, time, and charging efficiency. For each slot, 60 packets (i.e., one packet per minute), each having eight bytes, were generated, and selected packets were transmitted via a Quectel BC95 NB-IoT module [46]. The NB-IoT module works at a typical voltage of 3.6 V, current of 230 mA in the transmission state and a data rate of 4 kbps. Thus, each transmission cost about 13 mJ. The battery capacity was 7.7 $\times 10^{3}$ J (1800 mAh).

The performance metrics included the objective function and failure rate. The objective function is sum(N)−var(N), and we refer to this value as utility for simplification hereafter. The failure rate is defined as the ratio between the decisions violating ENO and all decisions, which is evaluated to demonstrate the robustness of UHETS against the harvested energy randomness.

### 6.1. Prediction Model

Since the Gaussian distribution is built based on a prediction model of harvested energy, this prediction model has a big impact on the performance. In this subsection, we explore two prediction models for evaluation. One is EWMA and the other is a Weather Forecast (WF)-based prediction model [47].

EWMA predicts the amount of harvested energy during slot *t* on day *d* as:

(28) $${\hat{E}}_{h}^{d+1}\left(t\right)=w{\hat{E}}_{h}^{d}\left(t\right)+\left(1-w\right)E_{h}^{d}\left(t\right),$$

where $E_{h}^{d}\left(t\right)$ denotes the amount of energy harvested during slot *t* on day *d*, ${\hat{E}}_{h}^{d}\left(t\right)$ is the estimated amount, and *w* is a weighting factor. We set w=0.5 since it can yield the least prediction error [2].

**Figure 6:**
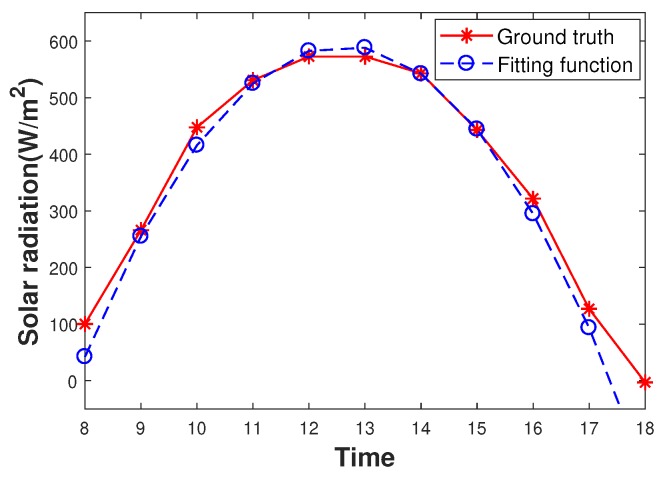
Fitting curve of the Weather Forecast (WF).

WF assumes the harvested solar energy for every slot can be formulated as a quadratic function and further influenced by the cloud coverage. Specifically, ${\hat{E}}_{h}\left(t\right)$ can be estimated as:

(29) $${\hat{E}}_{h}\left(t\right)=\left(a{\left(t-b\right)}^{2}+c\right)\times \left(1-sky\_condition\right),$$

where a,b,c are empirical parameters obtained from historical data and sky_condition is the percentage of the sky that is covered by cloud, which was obtained from the weather forecast station. The dataset of sky_condition was obtained from the National Weather Station (NWS) [48]. For every 3 h, NWS publishes a forecast for 3 h, 24 h, and 72 h. In our experiment, we utilized the forecast for 24 h as the decision period in UHETS is 24 h. Based on the meteorological data, a,b,c were obtained by minimizing the mean squared error, as shown in Table 2. Figure 6 shows the difference between the fitting curve by Equation (29) and the ground truth on a sunny day without cloud cover in January 2018.

Table 3 shows the comparison result between EWMA and WF in which the Mean Squared Error (MSE) represents the expected value of the square of the difference between the predicted values by EWMA and WF and the ground truth, and Root Mean Squared (RMS) is the the arithmetic square root of MSE. From Table 3, we can see that WF was more accurate than EWMA, the MSE of WF being roughly $1.09\times 10^{-3}$, while that of EWMA was $1.44\times 10^{-3}$.

### 6.2. The Impacts of Parameters

**Figure 7:**
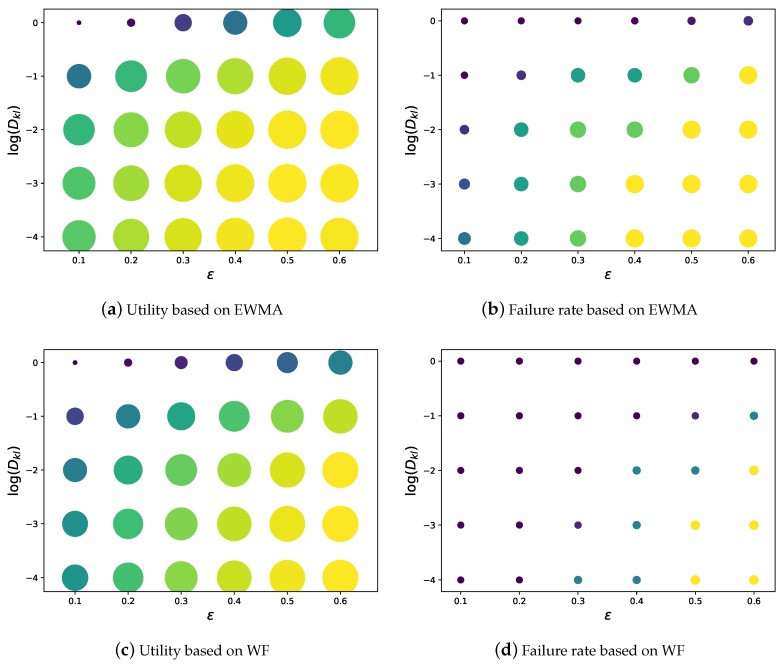
Impact of parameters ϵ and $D_{KL}$. We present the amount of utility in (**a**,**c**) and the failure rate in (**b**,**d**) using the size of each circle, respectively.

As mentioned in Section 3, the parameters ϵ, $D_{KL}$ indicate the robustness level against the harvested energy randomness. Thus, in Figure 7 we explored their impacts on the performance of UHETS based on the two prediction models described in Section 6.1.

Intuitively, the higher ϵ, the higher the tolerance on ENO violation, thus a higher failure rate and utility. Higher $D_{KL}$ indicates that we assumed the real-world PDF was more different from the Gaussian PDF and that UHETS will yield a stricter worst case bound. In this way, the failure rate and the utility will decrease with increasing $D_{KL}$. The experimental results were two-fold. Firstly, UHETS enabled flexible transmission scheduling, which traded between energy maintenance and utility. Secondly, by setting small $D_{KL}$ and ϵ, UHETS was robust against the harvested energy randomness. This conclusion holds for both UHETS based on EWMA and WF.

When we compared the performance between the two prediction models, we observed that with a similar utility, the failure rate achieved by WF was slightly lower than EWMA. This conforms to the expectation that WF is superior to EWMA in terms of prediction accuracy. Furthermore, we observed that in order to achieve the same utility, WF requires smaller $D_{KL}$ and higher ϵ. This is also because of the higher prediction accuracy of WF.

**Figure 8:**
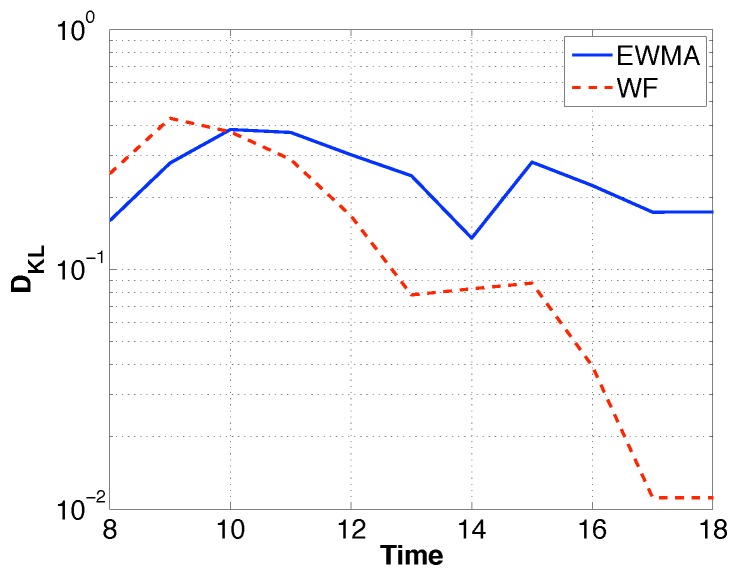
Empirical $D_{KL}$.

A more reasonable way is to set $D_{KL}$ empirically based on historical data. The empirical values of $D_{KL}$ were calculated according to the definition of KL divergence $D_{KL}=E_{f}\left[lnf\left(S_{h}\left(t\right)\right)-lng\left(S_{h}\left(t\right)\right)\right]$ where $f(S_{h}\left(t\right))$ was obtained from historical data and $g(S_{h}\left(t\right))$ was obtained as mentioned in Section 3.3.2. Among all the values of $D_{KL}$ for each day, we chose the maximum value as the final divergence limit. Figure 8 plots the empirical values of $D_{KL}$ by EWMA and WF. In the later experiments, we chose to set different $D_{KL}$ for each slot.

### 6.3. Performance

In this subsection, we compare the proposed UHETS with three approaches. The first approach is OPTworking as the benchmark. In OPT, the optimization problem is solved directly based on the ground truth data. Thus, it yields the optimal transmission scheduling that achieves the most transmissions with a zero failure rate. The second one solves the optimization problem based on EWMA. With a slight abuse of notation, we refer to this approach as EWMA hereafter. The third one is a Lower Bound-based Transmission Scheduling approach (LBTS). In LBTS, we adopt the energy model presented in [2], the harvested energy is characterized by a $\left(\rho ,\sigma_{1},\sigma_{2}\right)$ function, where ρ is the average rate of the harvested energy over a long duration, and the burstiness is bounded by $\sigma_{1}$ and $\sigma_{2}$. Specifically, the harvested energy until slot *t* is lower bounded by $\rho t-\sigma_{2}$. LBTS uses this lower bound of harvested energy in the energy constraints.

**Figure 9:**
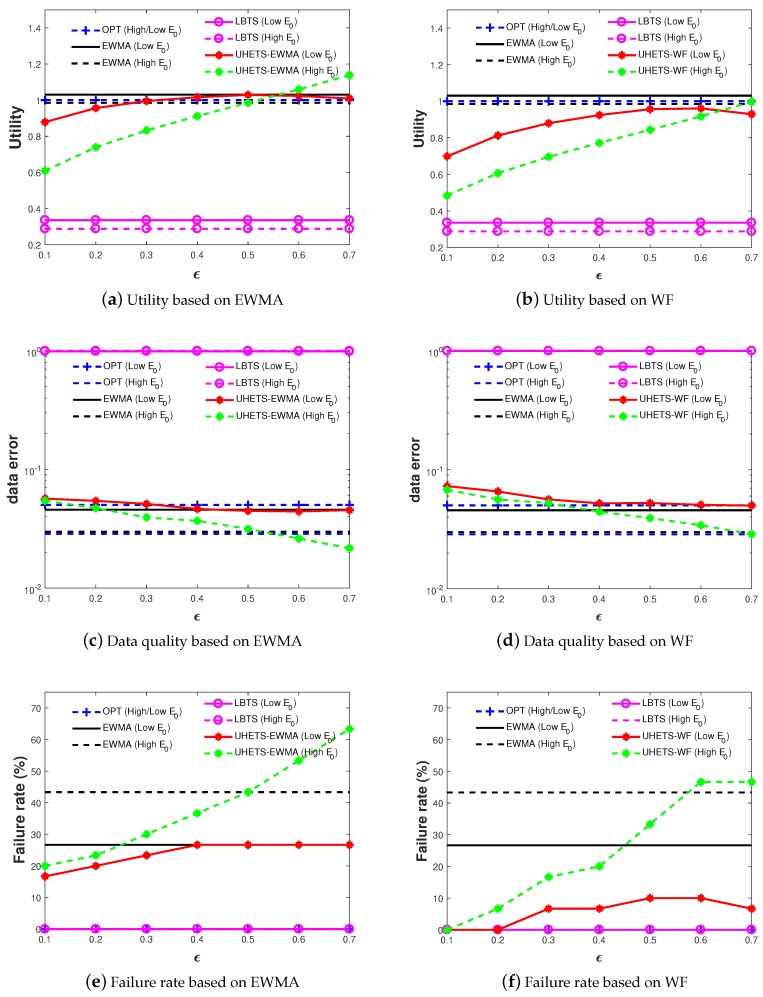
Statistic performance: comparison of Uncertain Harvested Energy-based Transmission Scheduling (UHETS) based on EWMA or WF with pure EWMA, Lower Bound-based Transmission Scheduling (LBTS), and OPT.

We evaluated the approaches in terms of failure rate, utility, and recovered quality in the case of tight initial battery energy (i.e., $E_{0}=2$ J in this paper) and lenient initial battery energy (i.e., $E_{0}=2\times 10^{3}$ J). Please recall that the failure rate is defined as the ratio between the decisions violating ENO and all decisions and utility is the objective function. Regarding utility, to straightforwardly illustrate the comparison results of different approaches, we show the ratio between the utility of different approaches and that of OPT in Figure 9. The recovered data quality was evaluated by the reconstruction error by using matrix completion, which is defined as:

$$e=||M-M^{\prime }{\left|\right|}_{2}/{\left||M|\right|}_{2},$$

where M denotes the original data matrix and $M^{\prime }$ is the reconstructed data matrix by using matrix completion. Since advanced matrix completion design is out of the scope of this paper, we used a classic matrix completion method SVT [38] for this purpose.

SVT recovers the original data matrix M by solving the following optimization problem:

(30) $$\begin{array}{cc} \min & {\tau ||X||}_{*}+\frac{1}{2}{\left||X|\right|}_{F}^{2} \end{array}$$

(31) $$\begin{array}{cc} s.t. & \Omega •X=\Omega •M \end{array}$$

where X is the recovered $M^{\prime }$, τ>0, ${\left||\;|\right|}_{*}$ denotes the nuclear norm, and ${\left||\;|\right|}_{F}$ is the Frobenius norm. Fix τ>0 and δ of the scalar step size, starting from $Y_{0}=0\in R^{n\times T}$, at the kth iteration, SVT induces:

(32) $$\begin{array}{c} X_{k}=D_{\tau }\left(Y_{k-1}\right) \end{array}$$

(33) $$\begin{array}{c} Y_{k}=Y_{k-1}+\delta \Omega •\left(M-X_{k}\right) \end{array}$$

until the stopping criterion is reached, where $D_{\tau }\left(*\right)$ is the soft-thresholding operator on the singular values of matrix *. When implementing SVT, we used the source code from [49] with parameters $\tau =10\sqrt{nT},\delta ={\left||N|\right|}_{1}/\left(10nT\right)$, maximum number of iterations $10^{3}$, and stopping criterion $\epsilon =10^{-4}$.

We firstly focus on the performance UHETS based on EWMA, as shown in Figure 9a,c,e.

In the case of tight initial battery energy, all approaches were expected to yield lower utility, consequently higher data recovery error and a lower failure rate, as well. This is because lower $E_{0}$ has a stronger impact on Constraint (2), while higher $E_{0}$ will impose a weak effect on (2). Our experimental results confirmed this expectation. As shown in Figure 9a, UHETS obtained a growing utility with increasing ϵ and approached that of EWMA with a high ϵ. Consequently, the data error of UHETS decreased and approached that of EWMA with increasing ϵ. We also observed that when ϵ exceeded 0.5, there was a turning point regarding both the utility and data error. This is because n(t) for t∈[0,8] was mainly determined by the tight initial energy. As ϵ increased, n(t) for the latter slots rose consequently, which will lead to the augmentation of var(N). As a result, the utility decreased slightly, as did the recovery data error. With slightly lower utility and data error as compared with EWMA, the failure rate of UHETS always stayed below the 28% achieved by EWMA. Among all, LBTS obtained a zero failure rate, but the lowest utility and data quality. This lower bound-based performance guarantee method imposes such an overly tight constraint that the utility degrades significantly, which makes LBTS impractical to use in reality. We can conclude that UHETS was able to achieve flexible transmission scheduling and energy maintenance.

In the case of lenient initial battery energy, the utility would increase, data error decrease, and failure rate grow consequently as well for both UHETS and pure EWMA, while LBTS would remain unchanged. Specifically, the failure rate of EWMA was boosted to roughly 43%, while UHETS had varying failure rates from 18–43% under ϵ≤0.5.

UHETS based on WF as shown in Figure 9b,d,f showed similar experimental results except that the utility and data rate were inferior and the failure rate was superior to UHETS based on EWMA under the same ϵ.

In summary, by comparison of the experimental results in Figure 9, it was demonstrated that UHETS was capable of providing flexible service performance by tuning parameter ϵ.

### 6.4. UHETS-Adaptation

**Figure 10:**
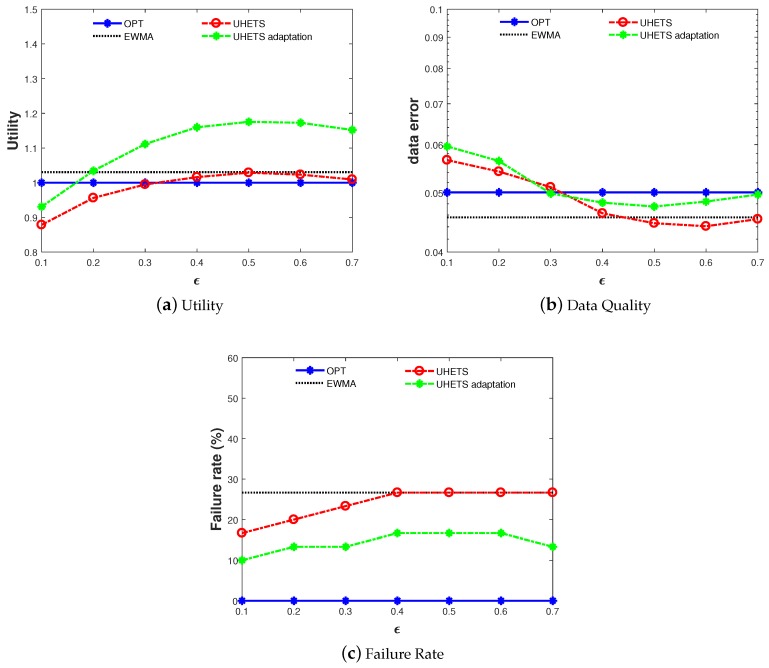
Performance of UHETS-adaptation.

This subsection compares the performance of UHETS-adaptation based on EWMA (as shown in Figure 10 to OPT, EWMA, and UHETS. We only plot the performance in the case of tight initial battery energy based on EWMA since the performance for other cases showed similar results. As shown in Figure 10a, by adaptively adjusting the transmission scheduling, the utility of UHETS-adaptation was improved greatly compared with UHETS. With increasing ϵ, the data error approached OPT and EWMA. Figure 10c clearly shows that the failure rate of UHETS-adaptation was lower than pure UHETS. This figure demonstrates that UHETS-adaptation was capable of both improving the utility and reducing the failure rate with lightweight extra computational overhead.

## 7. Conclusions

In this paper, we focused on the environment sensing IoT applications and aimed to address the utility maximization problem considering the randomness of the harvested energy. To handle the randomness gracefully, we firstly formulated the utility maximization problem subject to energy constraints with the harvested energy as a random variable. Profiling the harvested energy as a random process biased by a known distribution, we replaced the random variable with a constant so that the utility maximization problem could be solved using a traditional method. We also proposed an adaptive transmission scheduling approach that could adjust the transmission scheduling based on real-time harvested energy. Numerical experiment results demonstrated that UHETS was able to provide flexible transmission scheduling, which can achieve a good trade-off between ENO guarantee and utility maximization.

In this paper, data quality was implicitly optimized in the design of the transmission scheduling approach. In future work, we plan to directly use data quality as an explicit optimization objective, and the joint design of matrix completion and transmission scheduling has potential to further improve the data quality. Furthermore, as discussed in Section 5.2, the transmission failure caused by interference or channel failure is not considered in UHETS. We plan to design cross-layer transmission scheduling considering an interference and retransmission scheme in the future.

Moreover, we only focused on solar energy as the energy source. However, there is a rich body of energy sources such as vibration energy, wind energy, and so on, that are difficult to model with a known distribution. The application of UHETS with these energy sources still needs to be explored in the future.

## Figures and Tables

**Table 1:** Notations used in this paper.

Symbol	Definition
*T*	Decision period, T=24 h in this paper
t∈[1,T]	Slot
ΔT	Duration of a slot, ΔT=1 h in this paper
$E_{max}$	Battery capacity
$E_{0}$	Initial battery energy
$E_{t}$	Residual battery energy at slot *t*
$E_{c}$	Energy consumption for a single packet
$E_{h}\left(t\right)$	Harvested energy at slot *t*
f(*)	Real-world distribution
g(*)	Gaussian distribution
*n*	The number of high-resolution samples generated at each slot
n(t)	The number of samples transmitted in slot *t*
N	Transmission decision N=[n(1),n(2),⋯,n(T)]

**Table 2:** Parameters in WF.

	a	b	c
January	−25.88	12.61	592.21
February	−21.87	12.50	738.19
March	−23.89	12.70	818.22

**Table 3:** Comparison of the two prediction models.

	MSE	RSE
EWMA	$1.44\times 10^{-3}$	$3.80\times 10^{-2}$
WF	$1.09\times 10^{-3}$	$3.30\times 10^{-2}$

## Appendix A

We now prove that problems (19)–(21) is a convex optimization problem.

**Proof.** We let *y* be $S_{h}\left(t\right)$ and $y^{\prime }$ be $S_{h}^{u}\left(t\right)$ and rewrite (19)–(21) as follows:

(A1) $$\begin{array}{cc} \max_{f(y)} & \int_{0}^{+\infty }w\left(y,y^{\prime }\right)\cdot f\left(y\right)dy \end{array}$$

(A2) $$\begin{array}{cc} s.t. & \int_{0}^{+\infty }\left[\ln f\left(y\right)-\ln g\left(y\right)\right]f\left(y\right)dy\leq D_{KL} \end{array}$$

(A3) $$\begin{array}{c} \int_{0}^{+\infty }f\left(y\right)dy=1. \end{array}$$

The objective function (A1) and Constraint (A3) are affined with respect to f(y). Next, we show that Constraint (A2) is convex. We define a convex function f(y)=−lny. As we know, *f* and its perspective function *g* should be convex or concave at the same time (The readers can refer to [40,50] for a more detailed proof.), and its perspective function is the relative entropy of *y* and *z*, i.e.,

(A4) $$\begin{array}{c} g(y,z)=-z\;\ln(y/z)=z(\ln\;z-\ln\;y), \end{array}$$

which is convex as well. Then, we have that the KL divergence $\int_{y\in S}\left[\ln f\left(y\right)-\ln g\left(y\right)\right]f\left(y\right)dx$ between distribution f(y) and g(y) is convex in f(y) and also g(y). As a result, the constraint (A2) is convex with respect to f(y). Finally, we claim that the optimization problem is convex. □

## Appendix B

We let:

(A5) $$\begin{array}{c} P\left(S_{h}\left(t\right),f,\alpha ,\beta \right)=E_{f}\left[w\left(S_{h}\left(t\right),S_{h}^{u}\left(t\right)\right)-\beta -\alpha \ln\frac{f(S_{h}\left(t\right))}{g(S_{h}\left(t\right))}\right]. \end{array}$$

Then, the derivative of $P(S_{h}\left(t\right),f,\alpha ,\beta )$ with respect to *f* can be derived as:

$$\begin{array}{c} \frac{\partial P}{\partial f}=\lim_{t\to 0}\frac{1}{t}\left[P\left(f\left(S_{h}\left(t\right)\right)+t\cdot g\left(S_{h}\left(t\right)\right)\right)-P\left(f(S_{h}\left(t\right))\right)\right]\\ =\int_{0}^{+\infty }\left(w\left(S_{h}\left(t\right),S_{h}^{u}\left(t\right)\right)-\alpha \ln\frac{f(S_{h}\left(t\right))}{g(S_{h}\left(t\right))}-\beta -\alpha \right)g\left(S_{h}\left(t\right)\right)dS_{h}\left(t\right). \end{array}$$

Applying the Karush–Kuhn–Tucker condition, we have:

(A6) $$\begin{array}{ccc} w\left(S_{h}\left(t\right),S_{h}^{u}\left(t\right)\right)-\alpha \ln\frac{f(S_{h}\left(t\right))}{g(S_{h}\left(t\right))}-\beta -\alpha & = & 0 \end{array}$$

(A7) $$\begin{array}{ccc} \int_{0}^{+\infty }f\left(S_{h}\left(t\right)\right)dS_{h}\left(t\right) & = & 1 \end{array}$$

(A8) $$\begin{array}{ccc} E\left[\ln\frac{f(S_{h}\left(t\right))}{g(S_{h}\left(t\right))}\right]-D_{KL} & \leq & 0 \end{array}$$

(A9) $$\begin{array}{ccc} \alpha \left(D_{KL}-E\left[\ln\frac{f(S_{h}\left(t\right))}{g(S_{h}\left(t\right))}\right]\right) & = & 0 \end{array}$$

From (A6), the optimal distribution function can be expressed as follows:

(A10) $$\begin{array}{c} f^{*}\left(S_{h}\left(t\right)\right)=g\left(S_{h}\left(t\right)\right)\exp\left(\frac{w(S_{h}\left(t\right),S_{h}^{u}\left(t\right))-\beta }{\alpha }-1\right). \end{array}$$

The Lagrangian multipliers α,β in (A10) should be chosen properly such that Conditions (A7)–(A9) are satisfied. By substituting (A10) to Expressions (A7)–(A9), we can straightforwardly obtain (α,β) by solving the following:

(A11) $$\begin{array}{c} U\left(S_{h}^{u}\left(t\right)\right)e^{\beta /\alpha }+V\left(S_{h}^{u}\left(t\right)\right)e^{(1-\beta )/\alpha }=0, \end{array}$$

(A12) $$\begin{array}{c} V\left(S_{h}^{u}\left(t\right)\right)e^{(1-\beta )/\alpha }-\beta -\alpha \left(1+D_{KL}\right)=0, \end{array}$$

where $U\left(S_{h}^{u}\left(t\right)\right)=G\left(S_{h}^{u}\left(t\right)\right)e^{-1}$, $V\left(S_{h}^{u}\left(t\right)\right)=\left(1-G\left(S_{h}^{u}\left(t\right)\right)\right)e^{-1}$, and G(*) is the cumulative distribution of $g(S_{h}\left(t\right))$.

## Appendix C

We use binary search and Newton’s method to seek a proper solution of $S_{h}^{u}\left(t\right),\alpha^{*},\beta^{*}$ as shown in Algorithm A1. The search range of $S_{h}^{u}\left(t\right)$ is [0,γ], and iteration tolerance is ρ. Initially, we started the search with lower bound l=0 and upper bound u=γ (*u* must be chosen large enough such that $P_{m}^{u}\left(u\right)>\epsilon$). We need to check if $P_{m}^{u}\left(m\right)>\epsilon$ or $P_{m}^{u}\left(m\right)<\epsilon$ where m=(l+u)/2 and halve the search radius as shown in Line 23 and Line 25, respectively. During each search, we use Newton’s method as seen in Lines 10–17 to calculate $P_{m}^{u}\left(m\right)$ such that Equations (A11) and (A12) are satisfied. The binary search terminates when the upper bound *u* converges to the lower bound *l*.

**Algorithm A1** The iterative method to calculate $S_{h}^{u}\left(t\right),\alpha^{*},\beta^{*}$
**Input:**$g(S_{h}\left(t\right))$, search range [0,γ], iteration tolerance ρ, ϵ. **Output:**$S_{h}^{u}\left(t\right)$. 1: l←0,u←γ // initialize the search range as [l,u]=[0,γ] 2: $H_{1}\left(S_{h}^{u}\left(t\right),\alpha ,\beta \right)\leftarrow U\left(S_{h}^{u}\left(t\right)\right)e^{\beta /\alpha }+V\left(S_{h}^{u}\left(t\right)\right)e^{(1-\beta )/\alpha }$, 3: $H_{2}\left(S_{h}^{u}\left(t\right),\alpha ,\beta \right)\leftarrow V\left(S_{h}^{u}\left(t\right)\right)e^{(1-\beta )/\alpha }-\beta -\alpha \left(1+D_{KL}\right)$, 4: $H\left(S_{h}^{u}\left(t\right),\alpha ,\beta \right)\leftarrow {\left[H_{1}\left(S_{h}^{u}\left(t\right),\alpha ,\beta \right),H_{2}\left(S_{h}^{u}\left(t\right),\alpha ,\beta \right)\right]}^{T}$ 5: **while**|u−l|>ρ**do** 6: //Until the search range converges 7: k←1 8: m←(l+u)/2 //*m* is the middle of the search range 9: $\alpha_{1}=2$, $\beta_{1}=-2$ //choose a random start for α,β 10: **while** $H(m,\alpha_{k},\beta_{k})>\rho$ **do** 11: //Newton iteration to calculate $\alpha^{*}$ and $\beta^{*}$ such that $H(m,\alpha^{*},\beta^{*})\approx 0$ 12: evaluate H(m,α,β) and Jacobian matrix J(m,α,β) 13: solve $J\left(m,\alpha ,\beta \right){\left[\Delta \alpha_{k},\Delta \beta_{k}\right]}^{T}=-H\left(m,\alpha ,\beta \right)$ 14: $\alpha_{k+1}\leftarrow {\left[\alpha_{k}+\Delta \alpha_{k}\right]}^{+}$ //update α 15: $\beta_{k+1}\leftarrow \beta_{k}+\Delta \beta_{k}$ //update β 16: k←k+1 17: **end while** 18: //now $H(m,\alpha_{k+1},\beta_{k+1})\leq \rho$ 19: $\alpha^{*}\leftarrow \alpha_{k+1}$ 20: $\beta^{*}\leftarrow \beta_{k+1}$ 21: $P_{m}^{u}\left(m\right)\leftarrow \alpha^{*}D_{KL}+\beta^{*}$ //update $P_{m}^{u}\left(m\right)$ using $\alpha^{*}$ and $\beta^{*}$ 22: **if** $P_{m}^{u}\left(m\right)<\epsilon$ **then** //if $P_{m}^{u}\left(m\right)<\epsilon$ 23: l←m //halve the search range to [m,u] 24: **else** 25: u←m //halve the search range to [l,m] 26: **end if** 27: **if** $|P_{m}^{u}\left(m\right)-\epsilon |<\rho$ **then** // If $P_{m}^{u}\left(m\right)\approx \epsilon$, the binary search terminates. 28: break 29: **end if** 30: **end while** 31: $S_{h}^{u}\left(t\right)\leftarrow m$ // $S_{h}^{u}\left(t\right)$ such that $P_{m}^{u}\left(S_{h}^{u}\left(t\right)\right)=\epsilon$ is obtained.
